# Protein crystal nucleation in pores

**DOI:** 10.1038/srep35821

**Published:** 2017-01-16

**Authors:** Christo N. Nanev, Emmanuel Saridakis, Naomi E. Chayen

**Affiliations:** 1 Rostislaw Kaischew Institute of Physical Chemistry, Bulgarian Academy of Sciences, Acad. G. Bonchev Str. Bl. 11, 1113 Sofia, Bulgaria; 2 Structural and Supramolecular Chemistry Laboratory, Institute of Nanoscience and Nanotechnology, National Centre for Scientific Research “Demokritos”, Athens 15310, Greece; 3 Computational and Systems Medicine, Department of Surgery and Cancer, Faculty of Medicine, Imperial College London, London SW7 2AZ, UK

## Abstract

The most powerful method for protein structure determination is X-ray crystallography which relies on the availability of high quality crystals. Obtaining protein crystals is a major bottleneck, and inducing their nucleation is of crucial importance in this field. An effective method to form crystals is to introduce nucleation-inducing heterologous materials into the crystallization solution. Porous materials are exceptionally effective at inducing nucleation. It is shown here that a combined diffusion-adsorption effect can increase protein concentration inside pores, which enables crystal nucleation even under conditions where heterogeneous nucleation on flat surfaces is absent. Provided the pore is sufficiently narrow, protein molecules approach its walls and adsorb more frequently than they can escape. The decrease in the nucleation energy barrier is calculated, exhibiting its quantitative dependence on the confinement space and the energy of interaction with the pore walls. These results provide a detailed explanation of the effectiveness of porous materials for nucleation of protein crystals, and will be useful for optimal design of such materials.

Knowledge of the structures of proteins to near-atomic resolution is of prime importance for successful structure-guided (rational) drug design and for various other biotechnological applications. The most powerful and common method of protein structure determination is X-ray crystallography; however, obtaining the necessary protein crystals is a major stumbling block in that process. Nucleation of a new crystal is the crucial first stage in crystallization, therefore inducing and controlling nucleation has been a key topic of research in the field for many years. A promising method is to introduce a heterologous solid surface or particle that possesses nucleation-inducing properties into the supersaturated protein solution. Early nucleation inducing materials were based on epitaxy, non-specific electrostatic attraction of the protein molecules by oppositely charged surfaces or attempts to increase the contact area between the crystallization solution and the surface, but these approaches had limited success^1^,^2^,^3^,^4^,^5^.

In 2001, Chayen *et al*. introduced a new approach to protein crystallization, using porous materials with a distribution of pore sizes of the same order of magnitude as protein molecules^6^. The hypothesis was that the pores would entrap protein molecules, thereby encouraging them to aggregate in crystalline order. Porous silicon with average pore sizes of around 5–10 nm (estimated standard deviation 3 nm), the first in a series of porous nucleation agents, was tested on a variety of proteins of Stokes’ radii ranging from 2 to 5 nm: catalase, concanavalin A, lysozyme, a phycobiliprotein, thaumatin and trypsin. The crystallization trials included a selection of different common precipitating agents and a wide range of pH (4.6–8.4). Porous silicon was successful in inducing nucleation at conditions where the solution otherwise remained clear (metastable), leading to the growth of large single crystals of diffracting quality in five of the six proteins tested^6^. Following these positive results, a commercial porous glass (No. 7930, Corning Inc., USA)^7^ was tested and found to induce nucleation of lysozyme, thaumatin and apoferritin. Both porous materials were thus successful in inducing nucleation of several model proteins but they were not shown to affect any of the tested target proteins. Moreover, the porous silicon was only stable for several weeks after which it became oxidized, thereby leading to blockage of the pores and loss of its ability to induce nucleation.

In the search for a more stable and widely effective porous nucleating agent, a Bioglass with pore sizes of 2–10 nm was designed and tested with model and target proteins. In the presence of the Bioglass, crystals of all seven proteins initially tested were obtained at metastable conditions, whereas no crystals were formed in control trials containing the same conditions without Bioglass. Again, the nucleant was effective at a range of pH values, lower (lysozyme, thaumatin), higher (c-phycocyanin, α-crustacyanin), or approximately equal (trypsin, myosin-binding protein-C) to the protein isoelectric points^8^. Further target proteins such as a beta lactamase^9^, a multidrug resistance protein (a membrane protein), alpha-actinin actin binding protein, as well as octakis(6-guanidino-6-deoxy)-γ-cyclodextrin^10^, were successfully crystallized on the Bioglass. Over the years, further porous materials with a distribution of pore sizes, such as carbon nanotubes^11^ and porous gold^12^, were found to induce nucleation of a variety of model and target proteins including lysozyme, thaumatin, trypsin, haemoglobin, myosin binding protein C and NSP9. Other porous materials such as (alumino) silicates of uniform pore sizes up to 5 nm (VPI-5 and MCM-41) were also investigated but were not found to influence crystallization^6^.

Thus, several porous materials, in particular Bioglass (Naomi’s Nucleant’), proved to be very successful in producing high quality crystals of both model and target proteins (Fig. 1). The discovery of the effectiveness of porous materials as nucleants for protein crystallization has set a trend for using such materials to induce nucleation (e.g. refs 7, 11, 12, 13, 14, 15, 16, 17).

Monte Carlo computer simulations have been used to study the effect of pore shape, size and interaction strength of the molecules with pore walls. Page and Sear^18^ have shown that nucleation is orders of magnitude faster at the pores than on a smooth surface. They found that both the size and shape of the pore strongly affect the nucleation rate. Supporting the conclusion that a pore can be more effective than a planar wall with the same affinity for a molecule, van Meel *et al*.^19^,^20^ also argued that a two-step vapour–crystal nucleation mechanism should act in pores.

Protein crystal nucleation in pores resembles capillary condensation to a great extent, but differs in its molecular-kinetic mechanism. Both obey general thermodynamics, according to which the decreased energy barrier for condensation (respectively crystal nucleation) is responsible for these phenomena. Their similarity is due to the fact that both occur in the metastable zone, i.e. at lower supersaturation values than homogeneous nucleation. Moreover, they are both highly dependent on pore radius and geometry. The difference is that capillary condensation is due to an increased number of van der Waals interactions between molecules inside the confined space of a capillary, leading to multilayer adsorption, while protein crystal nucleation in pores results from protein molecule diffusivity (which is the only mechanism of matter transport in pores) and their adsorption; van der Waals interactions between protein molecules in solutions are restricted to very small distances, usually smaller than a protein molecular diameter.

## Results

While absent in bulk solution, a critical supersaturation sufficient for crystal nucleation onset may arise in pores^21^, provided the system is not too close to equilibrium. Because protein molecules in pores behave differently from those in the bulk solution, a gradual accumulation of protein in sufficiently narrow pores can result from the combination of two effects. Firstly, because diffusion is the sole mass-transfer mechanism working in pores (solution flow and convection being ineffective), the protein molecules become (almost) trapped inside the confined pore space. Secondly, due to translational Brownian motion, which is equally probable in all directions, the large protein molecules land on pore walls with a probability several times greater than their probability of escape. Provided there is some attraction between the protein molecule and the pore wall, the molecules remain adsorbed, at least temporarily. Diffusion-driven equilibration of the protein concentration between pore interior and bulk solution is thus hindered. The adsorption capacity of protein molecules at solid surfaces is high^22^ even when affinity is very moderate, because larger molecules are able to contact the surface at more sites. But even after being desorbed, the protein molecules remain trapped inside the pores, while after desorption from a flat surface the molecules are free to return to bulk solution. In other words, although the protein molecules are desorbed from the pore walls just as frequently as from an equivalent flat surface, their probability of being re-adsorbed is much higher than in bulk solution due to the confined space. Reiterating this diffusion-adsorption scenario numerous times, protein molecules gradually accumulate in the pores.

### The dynamic adsorption-desorption process and diffusion displacement

Desorption, which is an escape of the molecules from the shallow adsorption potential well, is usually described, e.g. ref. 23, as an activated first-order rate process. Therefore, the desorption rate, R_d_ is written as:


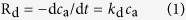


which, after integration, gives:


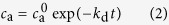


where *c*_a_ is the surface concentration of the adsorbed molecules and *t* is time, *c*_a_^0^ being the initial concentration of the adsorbed molecules.

The rate constant for desorption is *k*_d_ = *θ*exp (−E_d_/k_B_T)^23^, where E_d_ is the desorption activation energy, *θ* an “attempt frequency” for desorption, k_B_ is Boltzmann’s constant and T the temperature.

We may define a half-life τ_1/2_ for adsorption of protein molecules at the pore wall, as the time when *c*_a_ = *c*_a_^0^/2. Thus:


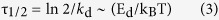


It has been found^24^ that the energy barrier, E_d_ for desorption of an isolated protein molecule is extremely low. The apparent activation energy is in the range of 2–4 kJ/mol, or less than 2k_B_T per molecule. Therefore, it has been observed that “regardless of the protein identity or surface chemistry, the vast majority of individual protein objects exhibited short residence times (<1 s)”^24^.

Our estimate of the size of a pore able to induce protein crystal nucleation due to adsorption being more frequent than desorption, is based on the mean squared diffusion displacement, <x^2^>:


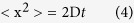


where D is the diffusion coefficient; a typical value for proteins is D_prot_ = 10^−6^ cm^2^ s^−1^.

This means that during adsorption time t = 0.5 s, a protein molecule can diffuse from wall to wall of a 10 μm sized pore (and adsorb there), the tacit assumption being that the molecule would follow the shortest path. However, because Brownian motion is equally probable in all directions, the escape probability of a protein molecule from the pore is about 1/6. Therefore, pores narrower than about 1μm can already accumulate protein. Note that such pores are still much larger than the typical nucleus size.

The total time during which the protein molecules are in the adsorbed state in such pores becomes thus greater than the total time during which they are in the desorbed state. Therefore, despite the increasingly higher amount of protein in the pores, no concentration gradient favoring back diffusion (from the pores towards bulk solution) can arise, at least temporarily. Thus, sufficiently narrow pores become something like “black holes” for macromolecules. Protein accumulation in pores can also be considered as a prerequisite for the formation of a denser, often metastable fluid phase, which initiates a two-step crystal nucleation in pores^25^.

In the following, we will look more closely at the two effects: (1) the increase in protein concentration resulting from Brownian motion inside the confined space of a pore, which we will simply call *space confinement effect*, and (2) the free energy-driven interaction of the protein molecules with the pore walls, which we will call *wall contact effect*.

### Supersaturation increase resulting from Brownian motion in a confined pore space

We first need to identify the factors which determine a pore’s capacity to increase the supersaturation. Evidently, the narrower the pore, the smaller the protein molecule escape probability, and thus the higher the concentration increase in the pore. On the other hand, the pore opening is reached and the protein molecules enter the pore with the same probability with which they reach an equally large flat surface area, which means that smaller openings are less accessible. Thus, a Poisson distribution should provide a good fit to the supersaturation increase in the pore as compared with that in the bulk solution.

**Figure 2:**
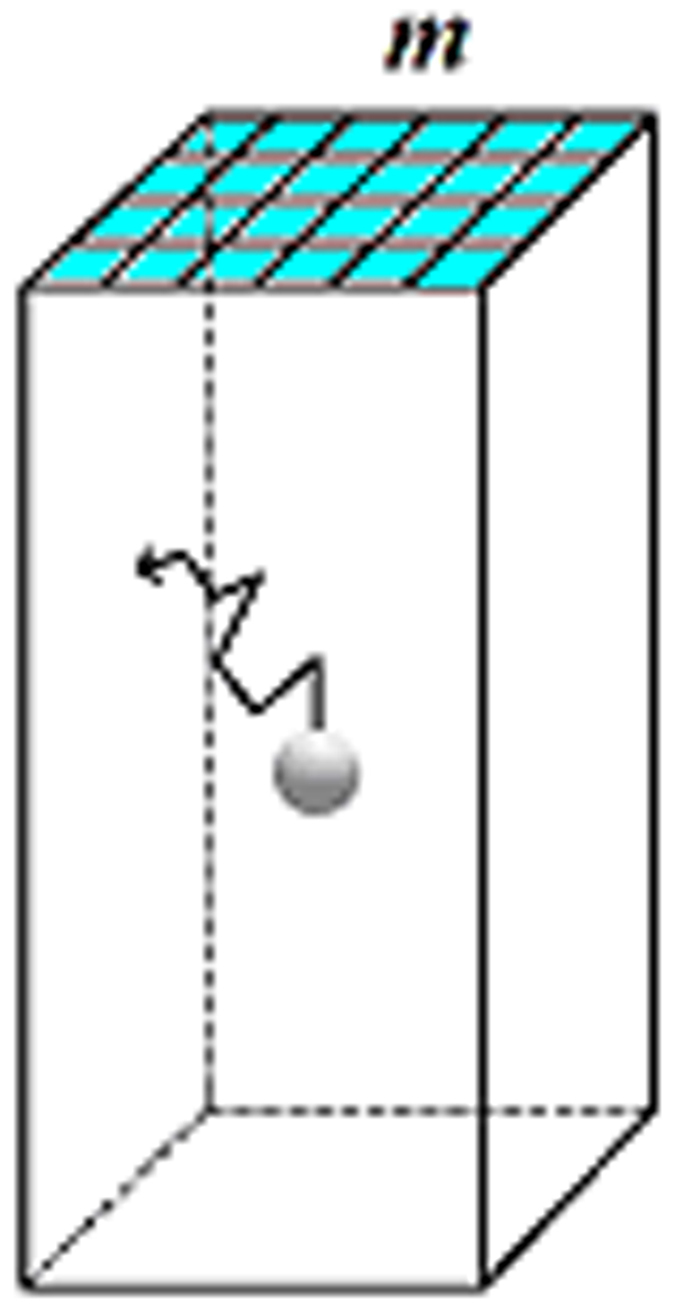
Simplest idealised pore-shape: a rectangular prism or cuboid. Pores in actual disordered porous materials such as Bioglass are much more complex, yet they tend to have analogous well-type morphologies. The pore is completely filled with solution. Flat pore walls prevent the nucleating crystal from trying to conform to any curved pore surface and becoming strained^40^. Crystal building blocks on the surface (i.e. in contact with the bulk solution) are highlighted in light blue.

Obviously, besides pore-opening size (expressed by the number, *m* of crystal building blocks forming the edge of a nucleus filling the entire pore cross section, Fig. 2), pore volume, *v,* and shape are also of importance. In intricate pores with many turns and corners, some protein molecules can be trapped quasi-permanently, or at least for a time that is sufficient for crystal nucleation onset. We thus introduce a geometric coefficient *g*, to account for the pore shape. To obtain the quantitative dependence of the chemical potential difference (supersaturation) Δ*μ* on *g* and *m*^2^, we multiply them by a factor *F*. Then, assuming a reverse proportionality of the difference between the supersaturation in a pore, Δ*μ*^*pore*^, and that in bulk solution, Δ*μ*^*bulk*^, on the pore size and volume, we have:


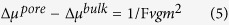


### Comparison of the in-pore with the homogeneous nuclear size

Let us denote by *n* the number of building blocks at the edge of a nucleus forming homogeneously at the same supersaturation in bulk solution. The ratio *m*/*n* can be calculated by applying the mean work of separation (MWS) method of Stranski–Kaischew^26^,^27^,^28^ for a Kossel crystal. This method operates by merely considering the relative bond energies between the individual building blocks in the crystal. The mean works of separation are calculated by counting the different kinds of bonds separately, and multiplying each number by the corresponding bond energy value; the products are then summed up and the result is divided by the total number of blocks in the corresponding crystal element (e.g. crystal face, ledge of growing crystal layer, etc). In the following, we will denote the mean works of separation by *φ*.

Only pores that are completely filled with solution are considered here. Only the front crystal face, filling the entire pore opening, participates in the molecular exchange with the parent phase. Its MWS is *φ*^*pore*^:


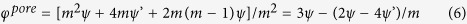


where *ψ* is the work (energy), which is performed for the separation of two building blocks of the crystal lattice, and *ψ’* the work of separation of one crystal building block from a cavity wall, *ψ’* ≈ E_d_.

On the other hand, the MWS, *φ*^*bulk*^, for the cubic face of the homogeneous nucleus formed in the parent phase under the same supersaturation, is^26^,^27^,^28^:


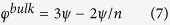


Equilibrium requires the equality *φ*^*pore*^ = *φ*^*bulk*^, which can be expressed as:


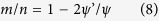


Denoting the adhesion energy (of crystal building block to wall) by *β*, the surface free energy (interphase crystal surface/solution energy) by *γ*, and the surface of the small cube which represents a building block in the Kossel crystal lattice^29^, *s*, we note that:

- Two crystal surfaces appear when two Kossel crystal lattice blocks are separated from each other, hence *ψ* ≈ 2*γs*.
- Only one crystalline surface appears when a Kossel crystal lattice block is separated from the cavity wall, hence *ψ’* ≈ *βs*. Replacing these values of *ψ* and *ψ’* in equation 8 we obtain:

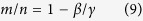


It is seen that the factor (1 − *β*/*γ*) is of prime importance. There are two extremes:

(a) *β* → 0, i.e. no adhesion, which means that *m* ≈ *n* and the pore does not play any role in the nucleation process.

(b) *β* ≈ *γ, m* ≈ 0, which means that nucleation in the cavity is spontaneous (there is no nucleation energy barrier at all). Thus, the closer *β* gets to *γ*, the more probable is crystal nucleation in pores. This result might explain why the tested Bioglass, which is a bioactive material attracting protein molecules, has proved very successful in inducing nucleation.

### Nucleation energy barriers from space confinement and interaction with pore walls

It is well known that the height of the nucleation energy barrier determines whether a crystal nucleus can form or not within a realistic time scale. Using the results from our MWS calculations, we separately show the impacts of the space confinement effect and the wall contact effect on protein crystal nucleation in pores.

The nucleation energy barrier is given by Classical Nucleation Theory (CNT)^29^ as a function of the volume of a crystal building block, *Ω* (usually the molecular volume in the crystal), the difference in chemical potentials (supersaturation) Δ*μ*, and surface free energy *γ*.

First taking into account the space confinement effect only (using equation 5), we can write the CNT-derived energy barrier for the Kossel crystal nucleus formation^29^ inside a pore:


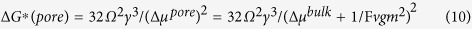


To express Δ*μ* in molecular-kinetic (Stranski-Kaischew) terms, we recall that the MWS is equal to the chemical potential taken with a negative sign, plus a substance and temperature dependent constant^30^. To adapt the MWS method, originally devised for a vapor phase-crystal system, to the more complex protein crystallization case it is necessary to add another constant.


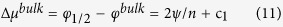


where *φ*_1/2_ is the work of separation of a molecule from the kink site and c_1_ is the protein and temperature-dependent constant.

Replacing into equation 10, the space confinement effect alone thus results in an energy barrier:


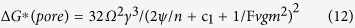


Now, denoting the energy barrier for homogeneous nucleation in the bulk solution (i.e. in the absence of pores) by ΔG*(*homo*), we obtain the ratio:


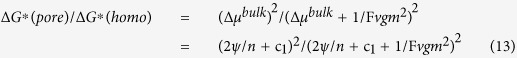


We note that ΔG*(*pore*)/ΔG*(*homo*) < 1, unless the pore volume is very high and/or the opening is very large. In such cases, ΔG*(*pore*)/ΔG*(*homo*) tends asymptotically to 1.

We must now consider the wall contact effect. Nucleation in pores is a type of heterogeneous nucleation (which is energetically preferred over homogeneous, because the nucleation energy barrier for the former is only a fraction of that for the latter). Therefore, recalling equation (6), the Δ*μ* value due to the wall contact effect is:


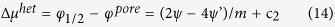


c_2_ being another constant.

It must be emphasized that the space confinement effect and the wall contact effect work synergistically. To combine both, we replace Δ*μ*^*bulk*^ with Δ*μ*^*het*^ in the energy barrier expression:


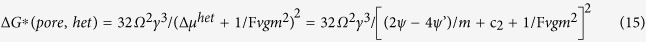


The ratio of the energy barrier arising from the combination of the two effects, to the homogeneous nucleation energy barrier is:


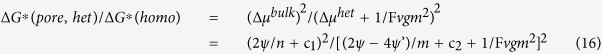


As expected, it is observed that:

When *β* → 0, i.e. *ψ*’ → 0, the only reduction of the energy barrier is due to the space confinement effect; the wall contact effect is absent.

When *β* ≈ *γ, m* ≈ 0, the size of the critical crystal nucleus is vanishingly small, therefore the energy barrier to nucleation disappears.

**Figure 3:**
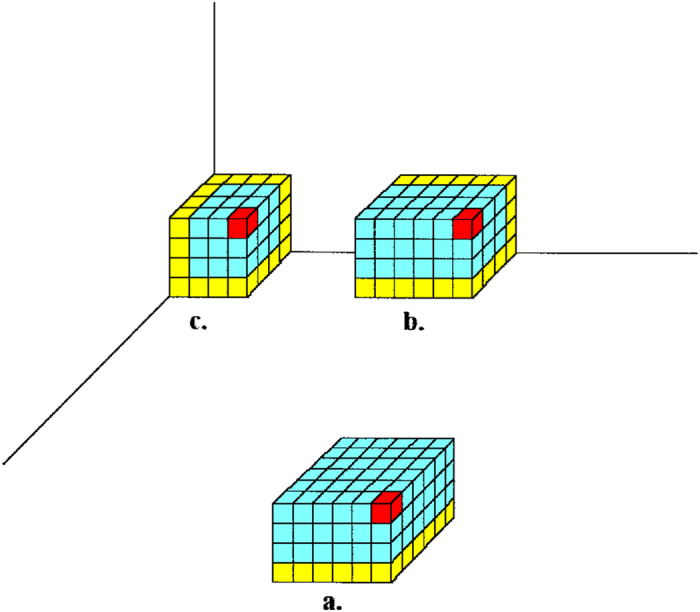
Heterogeneously formed crystals inside an idealised pore. (**a**) on the pore wall surface; (**b**) at concave edge joining two pore walls; (**c**) at concave corner of the pore. A single crystal building block at the apex of each nascent crystal is highlighted in red and the building blocks that are in contact with pore walls are highlighted in yellow.

We now consider the case in which the size of the pore opening is large enough to allow a critical nucleus smaller than the pore opening to form inside the pore. Crystal **c.** in Fig. 3 (which is easier to form than the other two crystals shown in the figure and, evidently, than the homogeneously nucleated one), is representative of this situation. This crystal possesses three equivalent faces, the MWS of which was calculated by Kaischew^31^. Denoting the number of molecules at one edge of the critical nucleus **c.** by *u*, and the pore opening size as previously, by *m*, we have:


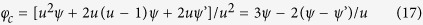


Equilibrium requires equality of all mean works of separation. Comparison of equations (6) and (17) shows that *m*/*u* < 1. Thus, the critical nucleus **c.** in Fig. 3 would be larger and therefore a smaller pore that is completely filled with the nucleus is more effective.

Introducing a third constant, c_3_:


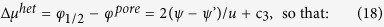



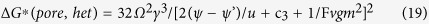


### Nucleation in partially filled cavities

When a porous material is immersed in a solution, it is possible that the latter does not penetrate the entire cavity, due to air remaining trapped at its bottom. In such cases, an air/solution interface is created, which should be enriched in protein molecules (see refs 32 and 33 for lysozyme). Because most proteins are surface active, an additional increase in local supersaturation, which favours nucleus formation, should be expected. However, as observed with insulin crystal nucleation^34^, the effect of the meniscus is negligible; minimum crystals were nucleated at the solution/glass/air three-phase angle. Most importantly, 2-dimensional nuclei are probably initially formed because, as they consist of a smaller number of molecules, they are energetically preferred. Subsequently, the 2D-nuclei further grow as 3D-crystals. The growth of 2D protein (ferritin) crystals at an air/solution interface is a well-known phenomenon^35^.

## Discussion

Our theoretical treatment explains the capacity of porous materials to induce protein crystal nucleation. We have demonstrated that two synergistic effects are at work, which may make porous materials very effective heterologous nucleants for macromolecular crystals. One is of geometric origin and is due to the trapping of protein molecules inside the confined space of a small pore, leading to a local increase in protein concentration. The other is due to free energy-driven adsorption of protein molecules at pore walls. From translational Brownian motion considerations, it is seen that the total time during which protein molecules are adsorbed on pore walls is greater than the time during which they are in the desorbed state, provided they have some affinity to the wall. These results provide a detailed explanation of the effectiveness of nucleants such as mesoporous Bioglass, as well as clues for the further optimisation of such nucleants. Our findings are applicable to all types of porous materials, provided the pore walls have some affinity for the protein molecules.

Based on equation 4, we have estimated an upper size limit for pores that may induce heterogeneous nucleation. We have shown that, for protein diffusion coefficients and energy barriers of desorption of single molecules as reported in the literature, pores of sizes less than about 1 μm can accumulate protein and thus induce a local concentration increase within the pore. But, although higher than the bulk solution concentration, that local concentration spike is not necessarily sufficient for nucleation induction. Comparison of equations 6 and 17 shows that the pores that are most effective in inducing nucleation are those of size nearest the critical nucleus size, which is generally a lot smaller than 1 µm. However, that nuclear size itself depends on the supersaturation, the energy of adhesion on pore walls, crystal surface free energy, and the size of the protein molecules, (see equation 9). Dependency on so many and fairly widely varying parameters indicates that the optimal porous material would possess a broad distribution of pore sizes, as it is indeed the case for Bioglass and for other successful porous materials. As pointed out in the Introduction, porous materials with more uniform pore diameters have also been tested and tend to be less successful nucleants. The pore sizes in the tested Bioglass have a Gaussian distribution with a maximum at 2–10 nanometres, but there are much larger (and smaller) pores too in the material. Sizes of protein crystal critical nuclei are usually at the higher end of that distribution, variously estimated to between 5 and 50 nm. For instance, insulin crystal nuclei consist of one to two hexamers, each 5 nanometres in size^34^. Although it was impossible to determine which pores the protein molecules actually ‘choose’ to nucleate at, these observations appear to corroborate our theoretical treatment.

In our theoretical treatment we have not considered the issue of protein denaturation upon adsorption on the pore surface. Protein denaturation is thought to be reversible for short residence times. For longer residence times, a slow denaturation process is thought to occur^36^, the extent of which is protein and surface-dependent^37^,^38^. In our model, we have assumed short residence times of the adsorbed proteins on the pore walls and small affinities, therefore a denaturation effect which may often be negligible. That denaturation upon adsorption is often either negligible or not disastrous for heterogeneous nucleation on foreign substrates is also shown by the large number of reported successes of heterologous nucleants, porous or not (uncontrolled heterogeneous nucleation on microscopic impurities or on the walls of crystallisation vessels is often considered as the main nucleation mechanism for protein crystals under ordinary crystallisation conditions)^29^. A possible situation is that the crystal layer that is immediately in contact with the foreign wall slowly denatures, by which time a stable crystal of properly folded molecules has had time to form upon it, whereas before criticality is achieved the short-lived adsorption events do not result in denaturation. It has also been proposed that denatured proteins may serve as nucleation centres^39^. It cannot however be excluded that in some cases, heterogeneous nucleation might indeed be prevented by this effect and this could be one of the reasons why heterogeneous nucleation attempts sometimes fail.

## Additional Information

**How to cite this article**: Nanev, C. N. *et al*. Protein crystal nucleation in pores. *Sci. Rep.* **7**, 35821; doi: 10.1038/srep35821 (2017).

**Publisher's note:** Springer Nature remains neutral with regard to jurisdictional claims in published maps and institutional affiliations.

## Figures and Tables

**Figure 1:**
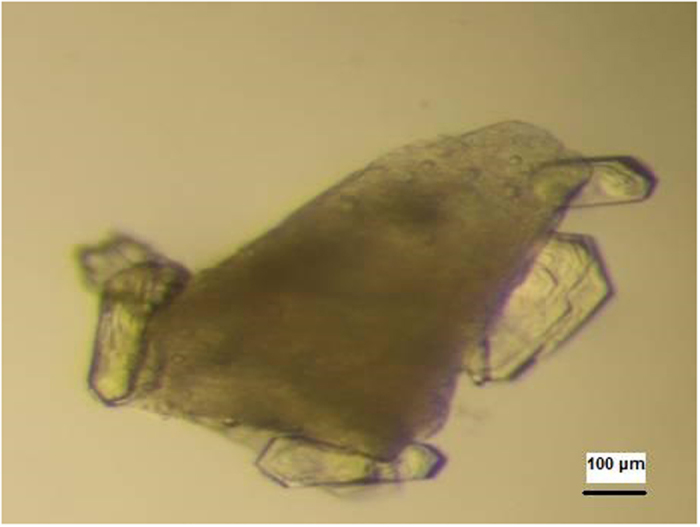
Crystals of a beta lactamase growing on Bioglass. The Bioglass appears as a spongy granule.

## References

[b1] McPherson A. & Shlichta P. Heterogeneous and epitaxial nucleation of protein crystals on mineral surfaces. Science 239, 385–387 (1988). 17836869 10.1126/science.239.4838.385

[b2] Falini G., Fermani S., Conforti G. & Ripamonti A. Protein crystallisation on chemically modified mica surfaces. Acta Crystallogr. D58, 1649–1652 (2002). 10.1107/s0907444902012763 12351879

[b3] Pechkova E. & Nicolini C. Protein nucleation and crystallization by homologous protein thin film template. J. Cell. Biochem. 85, 243–251 (2002). 11948680 10.1002/jcb.10123

[b4] D’Arcy A., Mac Sweeney A. & Haber A. Using natural seeding material to generate nucleation in protein crystallization experiments. Acta Crystallogr. D59, 1343–1346 (2003). 10.1107/s0907444903009430 12832806

[b5] Georgieva D. G., Kuil M. E., Oosterkamp T. H., Zandbergen H. W. & Abrahams J. P. Heterogeneous nucleation of three-dimensional protein nanocrystals. Acta Crystallogr. D 63, 564–570 (2007). 17452781 10.1107/S0907444907007810

[b6] Chayen N. E., Saridakis E., El-Bahar R. & Nemirovsky Y. Porous silicon: an effective nucleation-inducing material for protein crystallization. J. Mol. Biol. 312, 591–595 (2001). 11575916 10.1006/jmbi.2001.4995

[b7] Rong L., Komatsu H., Yoshizaki I., Kadowaki A. & Yoda S. Protein crystallization by using porous glass substrate. J. Synchrotron Radiat. 11, 27–29 (2004). 14646126 10.1107/s0909049503023525

[b8] Chayen N. E., Saridakis E. & Sear R. P. Experiment and theory for heterogeneous nucleation of protein crystals in a porous medium. Proc. Natl. Acad. Sci. USA 103, 597–601 (2006). 16407115 10.1073/pnas.0504860102 PMC1334630

[b9] Saridakis E. & Chayen N. E. Towards a ‘universal’ nucleant for protein crystallization. Trends Biotechnol. 27, 99–106 (2009). 19110330 10.1016/j.tibtech.2008.10.008

[b10] Khurshid S., Saridakis E., Govada L. & Chayen N. E. Porous nucleating agents for protein crystallization. Nat. Protoc. 9, 1621–1633 (2014). 24922271 10.1038/nprot.2014.109

[b11] Asanithi P. . Carbon-nanotube-based materials for protein crystallization. ACS Appl. Mater. Interfaces 1, 1203–1210 (2009). 20355914 10.1021/am9000858

[b12] Kertis F. . Heterogeneous nucleation of protein crystals using nanoporous gold nucleants. J. Mater. Chem. 22, 21928–21934 (2012).

[b13] Saridakis E. . Protein crystallization facilitated by molecularly imprinted polymers. Proc. Natl. Acad. Sci. USA 108, 11081–11086 (2011). 21690356 10.1073/pnas.1016539108 PMC3131372

[b14] Saridakis E. & Chayen. N. E. Polymers assisting in protein crystallization. Trends Biotechnol. 31, 515–520 (2013). 23764007 10.1016/j.tibtech.2013.05.003

[b15] Eisenstein M. The shape of things. Nat. Methods 4, 95–102 (2007).

[b16] Sugahara M., Asada Y., Morikawa Y., Kageyama Y. & Kunishima N. Nucleant-mediated protein crystallization with the application of microporous synthetic zeolites. Acta Crystallogr. D64, 686–695 (2008). 10.1107/S0907444908009980 18560157

[b17] Di Profio G., Curcio E., Ferraro S., Stabile C. & Drioli E. Effect of supersaturation control and heterogeneous nucleation on porous membrane surfaces in the crystallization of L-glutamic acid polymorphs. Cryst. Growth Des. 9, 2179–2186 (2009).

[b18] Page A. J. & Sear R. P. Heterogeneous nucleation in and out of pores. Phys. Rev. Lett. 97, 065701 (2006). 17026175 10.1103/PhysRevLett.97.065701

[b19] van Meel J. A., Liu Y. & Frenkel D. Mechanism of two-step vapour–crystal nucleation in a pore. Mol. Phys. 113, 2742–2754 (2015).

[b20] van Meel J. A., Sear R. P. & Frenkel D. Design principles for broad-spectrum protein-crystal nucleants with nanoscale pits. Phys. Rev. Lett. 105, 205501 (2010). 21231245 10.1103/PhysRevLett.105.205501

[b21] Stolyarova S., Saridakis E., Chayen N. E. & Nemirovsky Y. A model for enhanced nucleation of protein crystals on a fractal porous substrate. Biophys. J. 91, 3857–3863 (2006). 16920829 10.1529/biophysj.106.082545 PMC1630463

[b22] Dee K. C. An Introduction to Tissue-Biomaterial Interactions. 1–50 (John Wiley & Sons, 2002).

[b23] Atkins P. W. Physical Chemistry, *Fourth Edition*. (Oxford University Press, 1990).

[b24] Langdon B. B., Kastantin M. & Schwartz D. K. Apparent activation energies associated with protein dynamics on hydrophobic and hydrophilic surfaces. Biophys. J. 102, 2625–2633 (2012). 22713578 10.1016/j.bpj.2012.04.027 PMC3368127

[b25] Christenson H. K. Two-step crystal nucleation *via* capillary condensation. Cryst. Eng. Comm. 15, 2030–2039 (2013).

[b26] Stranski I. & Kaischew R. The theory of the linear rate of crystallisation. Z. Phys. Chem. A 170, 295–299 (1934).

[b27] Stranski I. & Kaischew R. Über den Mechanismus des Gleichgewichtes kleiner Kriställchen I. Z. Phys. Chem. B 26, 100–113 (1934).

[b28] Stranski I. & Kaischew R. Über den Mechanismus des Gleichgewichtes kleiner Kriställchen II. Z. Phys. Chem. B 26, 114–116 (1934).

[b29] Nanev C. N. Theory of nucleation In Handbook of Crystal Growth, Volume 1A, Fundamentals: Thermodynamics and Kinetics (ed. Nishinaga T.) 315–358 (Elsevier, 2015).

[b30] Kaischew R. Zur Theorie des Kristallwachstums. Z. Physik 102, 684–690 (1936).

[b31] Kaischew R. Commn. Bulg. Acad. Sci., Phys. Ser. (Bulgarian) 1, 100 (1950).

[b32] Douillard R. Kinetics of lysozyme adsorption at the air-buffer interface. Thin solid films 292, 169–172 (1997).

[b33] Hunter J. R., Kilpatrick P. K. & Carbonell R. G. Lysozyme adsorption at the air/water interface. J. Colloid Interface Sci. 137, 462–482 (1990).

[b34] Nanev Chr. N., Hodzhaoglu F. V. & Dimitrov I. L. Kinetics of insulin crystal nucleation, energy barrier, and nucleus size. Cryst. Growth Des. 11, 196–202 (2011).

[b35] Yoshimura H. Protein-assisted nanoparticle synthesis. Colloids Surf. A: Physicochem. Eng. Aspects 282–283, 464–470 (2006).

[b36] Soderquist M. E. & Walton A. G. Structural changes in proteins adsorbed on polymer surfaces. J. Colloid Interface Sci. 75, 386–397 (1980).

[b37] Nakanishi K., Sakiyama T. & Imamura K. On the adsorption of proteins on solid surfaces, a common but very complicated phenomenon. J. Biosci. Bioeng. 91, 233–244 (2001). 16232982 10.1263/jbb.91.233

[b38] Sharma S., Berne B. J. & Kumar S. K. Thermal and structural stability of adsorbed proteins. Biophys. J. 99, 1157–1165 (2010). 20712999 10.1016/j.bpj.2010.05.030 PMC2920629

[b39] Akella S. Characterizing Protein Crystal Nucleation. Ph.D. Thesis (Brandeis University, 2014).

[b40] Sear R. P. The non-classical nucleation of crystals: microscopic mechanisms and applications to molecular crystals, ice and calcium carbonate. Int. Mater. Rev. 57, 328–356 (2012).

